# Meclizine-induced enhanced glycolysis is neuroprotective in Parkinson disease cell models

**DOI:** 10.1038/srep25344

**Published:** 2016-05-05

**Authors:** Chien Tai Hong, Kai-Yin Chau, Anthony H. V. Schapira

**Affiliations:** 1 Department of Clinical Neurosciences, UCL Institute of Neurology, University College London, UK; 2 Department of Neurology, Shuang Ho Hospital, Taipei Medical University Taiwan

## Abstract

Meclizine is a well-tolerated drug routinely used as an anti-histamine agent in the management of disequilibrium. Recently, meclizine has been assessed for its neuroprotective properties in ischemic stroke and Huntington disease models. We found that meclizine protected against 6-hydroxydopamine-induced apoptosis and cell death in both SH-SY5Y cells and rat primary cortical cultures. Meclizine increases the level of 6-phosphofructo-2-kinase/fructose-2,6-biphosphatase 3 (PFKFB3), which activates phosphofructokinase, a rate-determining enzyme of glycolysis. This protection is therefore mediated by meclizine’s ability to enhance glycolysis and increase mitochondrial hyperpolarization. Meclizine represents an interesting candidate for further investigation to re-purpose for its potential to be neuroprotective in Parkinson disease.

Parkinson disease (PD) is the second most common neurodegenerative disease^1^. The pathological hallmark of PD is the degeneration of dopaminergic neurons in the midbrain substantia nigra (SN) with Lewy body deposition^2^. Clinically, PD leads to motor and non-motor symptoms, due to both dopaminergic and non-dopaminergic deficits that result in disability and a significant reduction in quality of life^3^. Although several of symptomatic treatments are available, there is no therapy able to slow disease progression.

Mitochondrial dysfunction is recognized as a significant feature of PD pathogenesis^4^,^5^,^6^. In addition to their role in bioenergetics, mitochondria are involved in mediating apoptosis. Several apoptotic markers, including Bax, caspase 9 and caspase-3 have been identified in SNpc of PD^7^,^8^,^9^,^10^. Increased reactive oxygen species (ROS) and depolarization of the mitochondrial membrane potential (ψm) are believed to trigger the intrinsic apoptotic pathway by increasing the mitochondrial outer membrane permeability (MOMP). Release of mitochondrial proteins including cytochrome c, takes place after MOMP and initiate the apoptotic cell death cascade^11^.

In the 1-methyl-4-phenyl-1,2,3,6-tetrahydropyridine (MPTP) and β-amyloid toxicity models, increased glycolysis has been suggested to be able to restore cellular ATP synthesis, control ROS production, and maintain ψm in order to protect cell death^12^,^13^,^14^,^15^,^16^,^17^. Meclizine, is a widely-used antiemetic, and has been shown to enhance glycolysis and protect against neuronal death in stroke and Huntington disease models^18^,^19^.

In the present study, we demonstrate the neuroprotective effect of meclizine in cell models of PD. Our data show that the protective mechanism of meclizine involves increased glycolysis without altering oxidative phosphorylation and total ATP levels, the maintenance of mitochondrial hyperpolarization and the inhibition of apoptosis. We found that meclizine enhances glycolysis by increasing the activity 6-phosphofructo-2-kinase/fructose-2,6-biphosphatase 3 (PFKFB3) activity, which mediates the synthesis of fructose 2,6-bisphosphate to activate phosphofructokinase.

## Results

### Meclizine protected 6-OHDA induced apoptosis and cell death in primary cortical neurons

**Figure 1:**
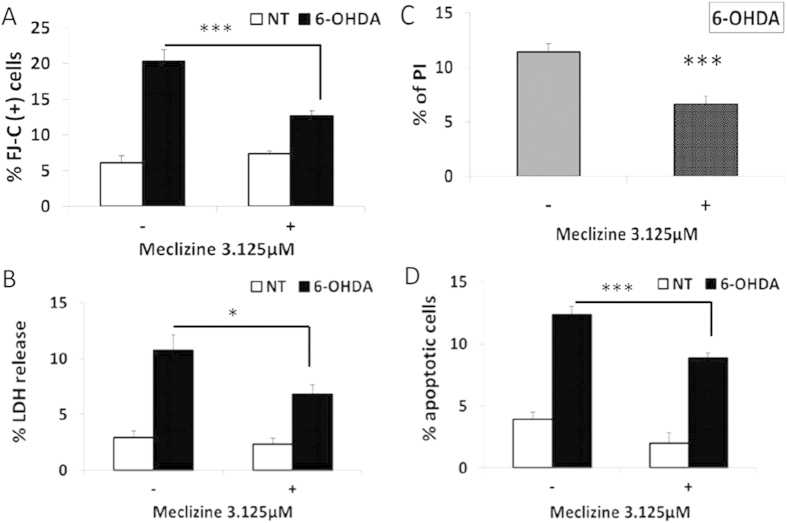
Meclizine protected 6-hydroxydopamine (6-OHDA) induced apoptosis and death in primary rat cortical cultures. (**A**) primary rat cortical cultures enriched with neurons, 3.125 μM meclizine treatment for 24 hours did not increase the spontaneous neuronal death but significantly reduced the percentage of FJ-C positive cells against 10 μM 6-OHDA treatment for 24 hours (control: 20.4 ± 1.6%, meclizine: 12.7 ± 0.7%, p < 0.001, n = 10). Meclizine was applied at the same time with 6-OHDA. (N.S., non-significant, ***p < 0.001). (**B**) In primary rat cortical cultures enriched with neurons treated with 10 μM 6-OHDA for 24 hours, 3.125 μM meclizine co-treatment significantly protected against cytotoxicity (control: 10.8 ± 1.4%, meclizine: 6.8 ± 0.9%, *p < 0.05, n = 8). Meclizine alone did not produce extra cell death. (**C**) The protection of meclizine against 10 μM 6-OHDA treatment on rat primary cortical culture cells for 24 hours was confirmed by PI binding assay (control: 11.5 ± 0.7%, meclizine: 6.6 ± 0.7%, p < 0.001, n = 10). Data were presented as mean ± S.E.M. and statistic analysis was performed by two-tailed Student’s t-test. (N.S., non-significant, ***p < 0.001). (**D**) In primary rat cortical cultures treated with 20 μM 6-OHDA for 6 hours, pre-treatment with 3.125 μM meclizine for 24 hours significantly decreased the percentage of apoptotic cells induced by 6-OHDA (control: 12.4 ± 0.6%, meclizine: 8.8 ± 0.4%, ***p < 0.001, n = 10).

The protection of meclizine was tested in primary rat cortical cultures highly enriched with neurons (supplementary S1). 6-OHDA induced a dose-dependent increase of Fluoro-jade C (FJ-C) stain, which reflected the neuronal death (supplementary S2A,B). The concentration of 10 μM 6-OHDA was chosen because of remarkable but not overwhelming effect of cell death (21.10 ± 5.37% FJ-C stained cells compared with no-toxin control: 6.65 ± 0.67% FJ-C stained cells). Compared with control, 3.125 μM of meclizine treatment, which was determined by the dose-dependent experiments of the protection of meclizine against 6-OHDA (supplementary S2C), for 24 hours did not increase the neuronal death detected by FJ-C stain. Upon 10 μM of 6-OHDA treatment for 24 hours, 3.125 μM meclizine significantly reduced the neuronal death release from 20.38 ± 1.57% to 12.68 ± 0.74% (p < 0.001) (Fig. 1A). The protection of meclizine was also confirmed by the LDH release assay: upon 10 μM of 6-OHDA treatment for 24 hours, 3.125 μM meclizine significantly reduced LDH release from 10.8 ± 1.4% to 6.8 ± 0.9% (p < 0.05) (Fig. 1B). Propidium iodide binding assay confirmed the protection by meclizine in primary rat cortical cultures (Fig. 1C).

To determine whether the prevention of cell loss by meclizine is an outcome of inhibiting apoptosis, we assessed caspase-3 activation upon 6-OHDA treatment. 6-OHDA is known to induce apoptosis and caspase-3 activation^20^. In primary rat cortical culture cells, apoptotic cells were identified by positive immunocytochemistry of cleaved caspase-3 (Supplementary S2D). In basal conditions, 3.125 μM of meclizine did not increase spontaneous apoptosis. Pre-treatment with 3.125 μM of meclizine for 24 hours before 20 μM of 6-OHDA for 6 hours significantly reduced the percentage of neurons with positive cleaved caspase-3 immunostaining from 12.4 ± 0.6% in the non-meclizine treated group to 8.8 ± 0.4% (p < 0.001) (Fig. 1D).

### Meclizine protected apoptosis and death in PD cellular model

**Figure 2:**
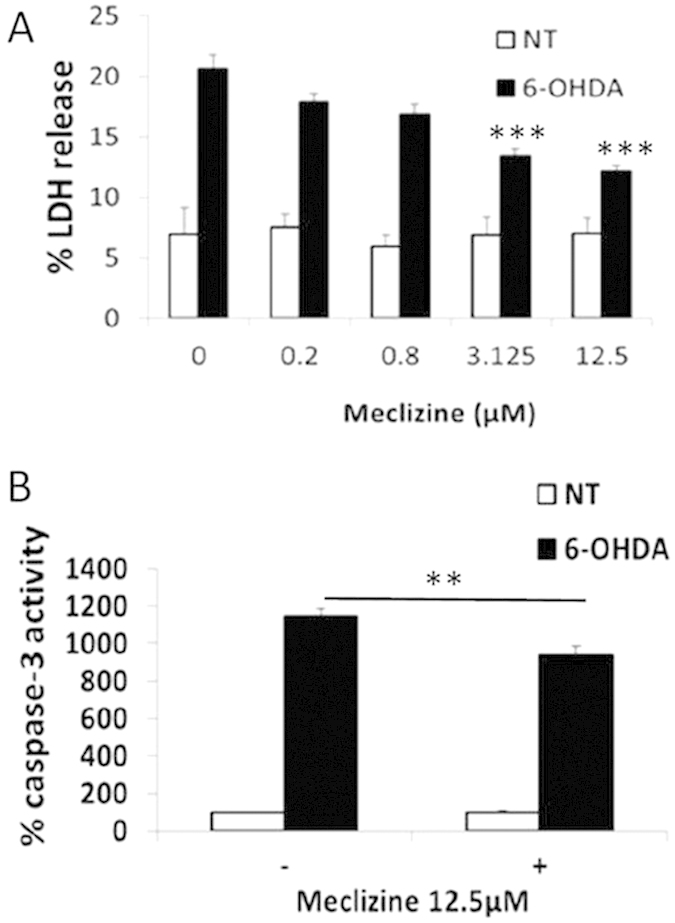
Meclizine protected apoptosis and cell death in PD cellular model. (**A**) In SH-SY5Y cells treated with 30 μM 6-OHDA for 48 hours, meclizine co-treatment exhibited a dose-dependent protection against cytotoxicity (as indicated by percentage LDH release). Meclizine at 3.125 μM and 12.5 μM produced significant protection (control: 20.6 ± 1.2%, 3.125 μM : 13.3 ± 0.6%, 12.5 μM:12.2 ± 0.4%, ***p < 0.001, n = 8). In addition, the maximum meclizine dose alone did not lead to cell death. (**B**) In SH-SY5Y cells treated with 100 μM 6-OHDA for 8 hours, pre-treatment with 12.5 μM meclizine for 24 hours significantly reduced 6-OHDA induced increased caspase-3 activity (control: 1148 ± 37%, meclizine:937 ± 47%, **p < 0.01, n = 7).

SH-SY5Y cells are widely used as a dopaminergic cell model and express the dopamine transporter and dopamine synthesis capacity. Meclizine treatment alone up to 12.5 μM for 48 hours did not induce cell death in SH-SY5Y cells. Upon 30 μM of 6-OHDA treatment for 48 hours, co-treatment with an increasing level of meclizine up to 12.5 μM produced a dose-dependent reduction of LDH release in SH-SY5Y cells; this became significant at 3.125 μM and 12.5uM, from 20.6 ± 1.2% to 13.3 ± 0.6 and 12.2 ± 0.4%% respectively (Fig. 2A). The anti-apoptotic effect of meclizine was also evaluated in SH-SY5Y cells. Meclizine treatment at 12.5 μM for 24 hours alone did not alter the caspase-3 activity. SH-SY5Y cells pre-treated with 12.5 μM of meclizine for 24 hours significantly down-regulated the activation of caspase-3 induced by 6-OHDA by 19% (Fig. 2B).

### Meclizine hyperpolarized mitochondria and prevented the depolarization induced by 6-OHDA

**Figure 3:**
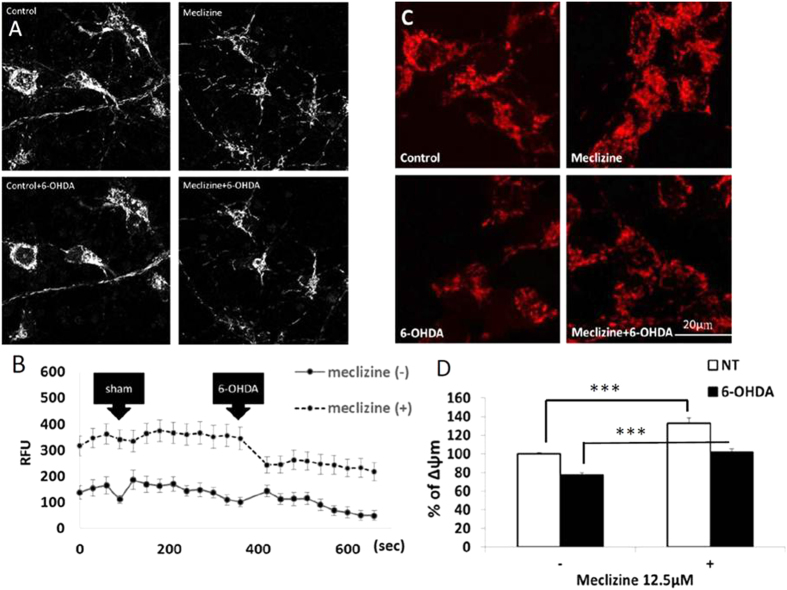
Meclizine hyperpolarized mitochondria and prevented 6-OHDA induced depolarization. (**A**,**B**) Representative images of TMRM staining on rat primary cortical neurons and the illustration of the change of net TMRM fluorescence intensity (RFU) under meclizine and 6-OHDA treatment. Rat primary cortical neurons pre-treated with 1 μM meclizine for 24 hours demonstrated remarkable higher net TMRM fluorescence intensity compared with control. Sham injection did not affect the net TMRM fluorescence intensity. 30 μM 6-OHDA was applied to the cells at 390 seconds and reduced the net TMRM fluorescence intensity was noted in both control and meclizine pre-treated neurons. However, meclizine pre-treated rat primary cortical neurons still exhibited higher net TMRM fluorescence intensity than the baseline of control neurons. Control and meclizine pre-treated TMRM staining images were acquired at 300 seconds and the 6-OHDAt-treated images in both conditions were acquired at 600 seconds. The net TMRM fluorescence was measured by the total TMRM fluorescence minus the background TMRM fluorescence. Data were presented as mean ± S.E.M. and the n = 5. (**C**,**D**) Representative images of TMRM staining of SH-SY5Y cells under different conditions and analysis of TMRM fluorescence intensity to produce a percentage of mitochondrial membrane potential (Ψm). Pre-treating SH-SY5Y cells with 12.5 μM meclizine for 48 hours significantly hyperpolarized mitochondria (control: 100.0 ± 1.5%, meclizine:132.8 ± 6.1%, ***p < 0.001, n = 10) and significantly prevented the mitochondrial depolarization induced by 100 μM 6-OHDA for 1 hour (control: 77.0 ± 2.8%, meclizine: 101.5 ± 4.0%, ***p < 0.001, n = 10).

6-OHDA causes increased ROS production and this can be measured as a significant fall in aconitase activity (see supplementary S3)^21^. Meclizine’s mechanism of action did not include any protection of aconitase, implying that it is not an anti-oxidant (supplementary S4). 6-OHDA also causes a fall in ψm and prevention of this is considered anti-apoptotic^12^,^22^. ψm was measured by TMRM fluorescence with adjustment for mitochondrial content. In rat primary cortical neurons, we found that 1 μM of meclizine treatment for 48 hours increased net fluorescence of TMRM compared with control, which reflects the mitochondrial hyperpolarization. Meanwhile, meclizine treatment on rat primary cortical neurons prevented 6-OHDA induced depolarization on rat primary cortical neurons, which would favour an anti-apoptotic action (Fig. 3A,B). In similar, we noticed that meclizine at 12.5 μM not only induced a 32% increase (p < 0.001) in ψm in SH-SY5Y cells, but also prevented 100 μM 6-OHDA treatment for one hour induced mitochondrial depolarization and maintained ψm at basal levels (Fig. 3C,D).

### Mitochondrial hyperpolarization and anti-apoptotic effect of meclizine are due to increased glycolysis

**Figure 4:**
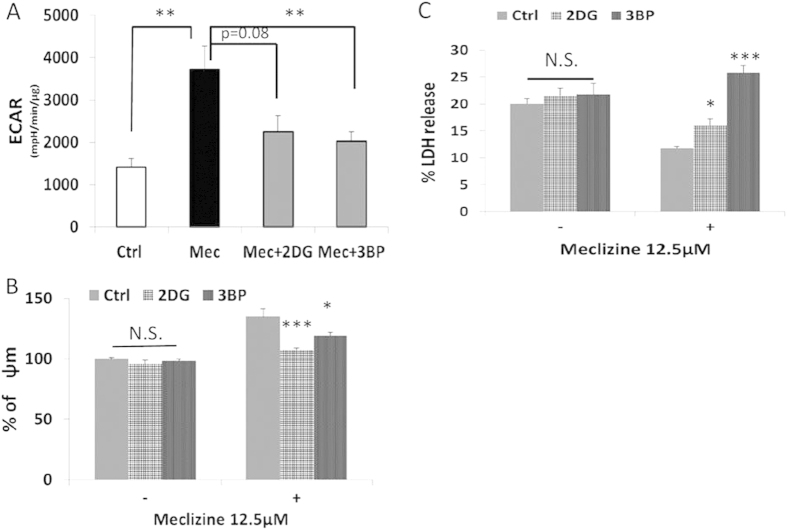
Mitochondrial hyperpolarization and prevention of cell death upon meclizine treatment in SH-SY5Y cells was resulted from increased glycolysis. (**A**) 12.5 μM meclizine for 48 hours significantly up-regulated the glycolytic activity as shown by extracellular acidification rate (ECAR) measured by XF analyzer. However, co-administration with glycolytic inhibitor, either 10 μM of 2-deoxy-glucose (2DG) or 5 μM of 3-Bromopyruvate (3BP) markedly reduced the enhancement of glycolysis. (control: 1405 ± 204, meclizine: 3613 ± 544, **p < 0.01; 2DG: 2359 ± 291, 3BP: 1856 ± 332 mpH/min/μg, p = 0.08 and 0.009, respectively, n = 6) (**B**) 10 μM of 2DG and 5 μM of 3BP alone did not affect the ψm. However, applying either glycolytic inhibitors significantly attenuated the mitochondrial hyperpolarization induced by 12.5 μM meclizine for 48 hours (meclizine: 135 ± 6.2%, meclizine+2DG: 107 ± 1.9%, meclizine+3BP: 119 ± 2.9%, ***p < 0.001 and *p < 0.05, respectively, n = 8). (**C**) Co-administration of either 10 μM of 2DG or 5 μM of 3BP, attenuated or reversed the protection of LDH release from meclizine against 30 μM 6-OHDA for 48 hours (meclizine: 11.8 ± 0.3%, meclizine+2DG: 16.0 ± 1.1%; meclizine+3BP: 25.8 ± 1.3%, *p < 0.05 and ***p < 0.001, respectively, n = 8).

SH-SY5Y cells treated with 12.5 μM meclizine for 48 hours significantly increased the glycolytic activity as measured by extracellular acidification rate, by 157% (p < 0.01) (Fig. 4A). This was confirmed by the use of glycolytic inhibitors 2-deoxy-D-glucose (2DG) and 3-bromopyruvate (3BP) that blocked the meclizine-induced increase in glycolysis (Fig. 4A). The increase in glycolysis did not affect oxidative phosphorylation or total ATP levels (supplementary S4).

10 μM of 2DG or 5 μM of 3BP prevented the mitochondrial hyperpolarization seen with meclizine treatment (Fig. 4B). Lastly, we investigated whether the meclizine-mediated neuroprotection against 6-OHDA is glycolysis-dependent in SH-SY5Y cells. LDH release measurements showed that 10 μM of 2DG or 5 μM of 3BP did not alter the cytotoxicity produced by 6-OHDA at 30 μM treated for 48 hours. In line with the data shown in Fig. 1A, co-treatment with 12.5 μM meclizine significantly decreased the LDH release induced by 6-OHDA. However, co-administration with the glycolytic inhibitors resulted in significant attenuation of protection against 6-OHDA (Fig. 4C).

### Meclizine enhanced glycolysis through increased levels of PFKFB3

**Figure 5:**
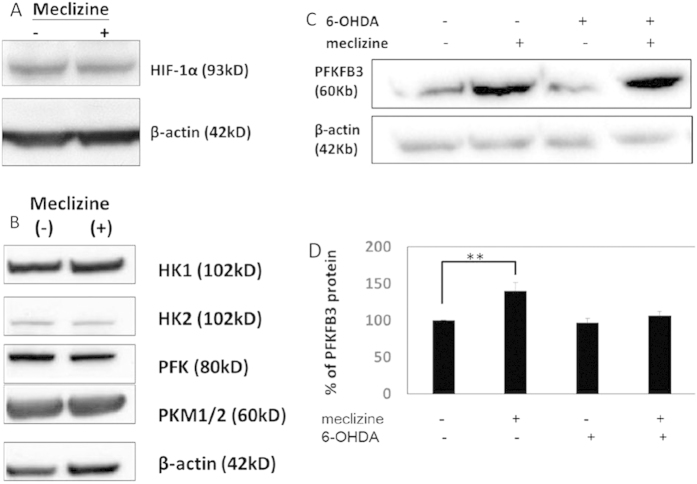
Increased glycolysis upon meclizine treatment was associated with increased PFKFB3 in SH-SY5Y cells. (**A**) Representative Western blot images demonstrate that meclizine treatment at 12.5 μM for 48 hours did not increase the protein level of hypoxia-inducible factor 1α (HIF-1α). (**B**) Representative Western blot images support that meclizine treatment at 12.5 μM for 48 hours did not alter the protein level of certain key glycolytic enzymes, including hexokinase 1 (HK1), hexokinase 2 (HK2), phosphofructokinase (PFKP), and pyruvate kinase isozymes M1/M2 (PKM1/2). (**C**,**D**) Representative Western blot images and densitometry analysis revealed that 12.5 μM meclizine treatment for 48 hours significantly increased the protein level of 6-phosphofructo-2-kinase/fructose-2,6-biphosphatase 3 (PFKFB3) in SH-SY5Y cells (139.5 ± 12.3%, p < 0.01). 100 μM 6-OHDA treatment for 6 hours on control SH-SY5Y cells resulted in a slight reduction of PFKFB3 protein level (96.3 ± 6.0%). Pre-treatment of meclizine prevents the reduction of PFKFB3 protein level caused by 6-OHDA (106.2 ± 5.5%). The protein expression amount was normalized by control SH-SY5Y cells. Data were presented as mean ± S.E.M. and the n = 6, **p < 0.01.

An increase in glycolysis may be HIF-1α-dependent, but levels of this protein were not increased with meclizine treatment (Fig. 5A). Alternatively, increased expression of glycolytic enzymes can also enhance glycolysis, but we found that 12.5 μM meclizine treatment for 48 hours did not alter the protein levels of: HK1, HK2, PFK and PKM1/2 (Fig. 5B).

PFKFB is a bifunctional enzyme that can catalyze the conversion of fructose-6-phosphate to fructose 2,6-bisphosphate (F2,6 P_2_) and vice versa. PFKFB Isoform 3 predominately effects the synthesis of F2, 6 P_2_, which is a powerful allosteric activator of PFKP and glycolysis^23^. 12.5 μM meclizine treatment for 48 hours significantly increased the protein level of 6-phosphofructo-2-kinase/fructose-2,6-biphosphatase 3 (PFKFB3) in SH-SY5Y cells (139.5 ± 12.3%, p < 0.01). 100 μM 6-OHDA treatment for 6 hours on control SH-SY5Y cells resulted in a reduction of PFKFB3 protein level (96.3 ± 6.0%) while pre-treatment of 12.5 μM meclizine for 48 hours prevented the reduction of PFKFB3 protein level caused by 6-OHDA (106.2 ± 5.5%) (Fig. 5C,D). These effect was seen on rat primary cortical neurons as well (Supplementary S5).

## Discussion

The present study demonstrates that meclizine, a glycolysis-enhancing agent, protects neuronal death in the 6-OHDA cell toxicity model. Meclizine hyperpolarizes mitochondria to prevent 6-OHDA induced apoptosis and mitochondrial depolarization and these effects are reduced by inhibition of meclizine-induced glycolysis. Meclizine increases glycolysis through increased levels of PFKFB3, which activates the key enzyme, PFK to enhance glycolysis.

Glycolysis is less efficient for ATP synthesis than oxidative phosphorylation. Neurons predominantly rely on oxidative phosphorylation for ATP generation and are not able to up-regulate glycolysis under respiratory inhibition^24^. However, it has been reported that glycolysis is responsible for neuronal vesicle transport in axonal regions^25^. In the present study, meclizine increases glycolysis but does not induce apoptosis and cell death in neuronal cells, which also support the notion that increasing glucose metabolism by glycolysis does not compromise the neuronal cells.

In the present study, meclizine increased cellular glycolysis, hyperpolarized mitochondria and protected against 6-OHDA toxicity. These effects were prevented by glycolysis inhibitors. Although hyperpolarization is also able to result from complex V inhibition, it is unlikely that meclizine hyperpolarizes mitochondria through this mechanism because complex V inhibition blocks oxidative phosphorylation and is toxic to the cells, whereas we saw no cytotoxicity upon meclizine treatment.

6-OHDA induces oxidative damage and apoptotic cell death and is widely-used in experimental PD models. Mitochondrial depolarization is a universal and early phenomenon of 6-OHDA induced apoptosis^26^,^27^,^28^. Meclizine enhanced glycolysis by increasing levels of PFKFB3 and protects against the fall in ψm induced by 6-OHDA treatment and initiation of the caspase cascade.

We have demonstrated that the anti-emetic meclizine increases glycolysis by increasing the levels of PFKFB3. This shift in energy metabolism is able to protect against the fall in ψm induced by 6-OHDA and reduce apoptotic cell death. We suggest that these results support further evaluation and potential re-purposing of meclizine as a modulator of energy metabolism for neuroprotection in PD.

## Material and Methods

### Cell models and treatments

SH-SY5Y cells were maintained as previously described^29^ with reagents supplied from Life Technologies (Paisley, UK). The method for setting up E18 primary rat cortical cultures enriched with neurons was adapted from previous well-established protocols^30^, and they were used 7 days post-culture. Meclizine was obtained from 2 independent sources namely Tocris Bioscience (Bristol, UK) and Santa Cruz Biotechnology(Heidelberg, Germany), and was dissolved in DMSO to produce a 12.5 mM stock. 6-hydroxydopamine (6-OHDA) was purchased from Sigma-Aldrich (Dorset, UK).

### Protein analysis

Whole cell lysates for Western blot analysis were prepared in 10 mM Tris–HCl pH 7.5, 0.1% of SDS and in the presence of protease inhibitors (Fisher Scientific, Loughborough, UK), followed by DNAse I digestion (Promega, Southampton, UK). Protein samples were separated under reducing conditions by SDS-PAGE using the Novex system (Life Technologies), transferred to PVDF (Millipore, Watford, UK) and were analyzed by a standard Western blot protocol using Amersham ECL Western Blotting Detection Reagent and Amersham Hyperfilm (GE Healthcare, Little Chalfont, UK). The following antibodies were used: anti-PFKFB3 antibody, anti-hypoxia inducible factor (HIF)-1α antibody, anti-phosphofrctokinase (PFK) antibody, anti-pyruvate kinase (PK) antibody, and hexokinase 1 and 2 antibody (HK1 & 2) (New England Biolabs, Hitchin, UK). Expression level of the above targets was corrected by the level of β-actin and normalized to non-treatment (NT) that is set as 100%.

Protein concentration was determined by bicinchoninic acid assay (BCA) according to manufacturer’s instructions (Fisher Scientific).

### Apoptosis and cell death analysis

Caspase-3 activity was measured from cell lysates according to the manufacturer’s instruction of the EnzChek^®^ Caspase-3 Assay Kit #2 (Life Technologies) which has been described previously^31^. The caspase-3 activity was corrected by the total protein level of the cell lysate and normalized to non-treatment (NT) that is set as 100%. Apoptotic cells in primary rat cortical culture were identified by immunocytochemistry using the anti-cleaved caspase-3 antibody (New England Biolabs) followed by Alexa Fluor-568 secondary antibody (Life Technologies), and mounted with Citifluor (London, UK) in the presence of DAPI. Fluorescent images were obtained by the Axioplan epifluorescent microscope (Zeiss, Cambridge, UK), from which positive stained and total number of cells were counted to produce a percentage of apoptotic cells. Lactate dehydrogenase (LDH) release assay (Roche, Burgess Hill, UK) was used to quantify the cell death and the protocols had been described^32^. The percentage LDH release was obtained from the experimental to the Triton X-100 treated total release. For the detection of neuronal death in primary culture, cells were stained with 0.001% Fluoro-Jade C (FJ-C)(Millipore) after fixed with 4% paraformaldehyde. Positive stained cells were counted under by epifluorescent microscope by examiner blinded to the condition.

### Ψm analysis

Maximal steady state mitochondrial fluorescence upon Tetramethylrhodamine, methyl ester (TMRM, Life Technologies) staining at 25 nM was measured from <1.5μm confocal images obtained from the Zeiss 510 laser scanning microscope after z-projection, as previously described^33^,^34^. Images obtained from SH-SY5Y cells were analyzed by ImageJ software (National Institutes of Health, Maryland, USA) following previously described analysis protocols^35^. Briefly, raw images were background corrected, linearly contrast optimized, applied with a 7 × 7 ‘top hat’ filter, subjected to twice 3 × 3 median filter, and then threshold, to generate binary images. Mitochondrial content per cell was estimated based on mitochondrial occupancy of these binary images. Mitochondrial fluorescence of each cell from the background corrected images was corrected by the mitochondrial content to produce the Ψm.

For the rat primary cortical neurons, net TMRM fluorescence is obtained by the total TMRM fluorescence minus the background TMRM fluorescence.

Oxygen consumption rate (OCR) and extracellular acidification rate (ECAR).

OCR and ECAR were measured by the XF extracellular flux analyzer following manufacturer’s instructions (Seahorse Bioscience, MA, US). For measuring OCR, 25 mM glucose, 1 μg/ml oligomycin and 2 μM rotenone were loading sequentially whereas for ECAR, 25 mM glucose, 1 μg/ml oligomycin and 100 μM 2DG were applied.

### Aconitase assay

Aconitase enzymatic activities were measured as described^36^, subsequently activity was corrected by protein amount.

### Total ATP measurement

Total ATP was measured using the ATP Bioluminesence Assay kit CLSII (Roche, Mannhein, Germany), from the cell lysates and that was corrected by the protein level.

### Statistics

All data were presented as mean ± standard error of mean (S.E.M.). Statistics was performed by either two-tailed Student’s t- test or one-way ANOVA with post-hoc analysis. *p* value less than 0.05 is considered as statistically significant.

## Additional Information

**How to cite this article**: Hong, C. T. *et al.* Meclizine–induced enhanced glycolysis is neuroprotective in Parkinson disease cell models. *Sci. Rep.* **6**, 25344; doi: 10.1038/srep25344 (2016).

## Supplementary Material

Supplementary Data
