# Supplementary material for: Meclizine-induced enhanced glycolysis is neuroprotective in Parkinson disease cell models
Source: Sci Rep. 2016 May 5;6:25344. doi: 10.1038/srep25344 (PMC4857109; doi:10.1038/srep25344)
Supplement: Supplementary Data [file srep25344-s1.doc]

**Title:** Meclizine-induced enhanced glycolysis is neuroprotective in Parkinson disease cell models

**Authors:** Chien Tai Hong^1,2^, Kai-Yin Chau^1^, Anthony HV Schapira^1, #^

**Affiliation:**

1. Department of Clinical Neurosciences, UCL Institute of Neurology, University College London, UK
2. Department of Neurology, Shuang Ho Hospital, Taipei Medical University

Taiwan

#Corresponding author

Proofs and reprint requests to: Anthony HV Schapira, 3B-94, Upper 3rd Floor

Institute of Neurology, Hampstead Campus. Rowland Hill St. London

NW3 2PF

e-mail: a.schapira@ucl.ac.uk

Tel: 020 7830 2012

Fax: 020 7472 6829

Key words: meclizine, glycolysis, Parkinson disease, mitochondria, apoptosis

Supplementary Data

S1. Identification the high purity of neurons in primary rat cortical culture


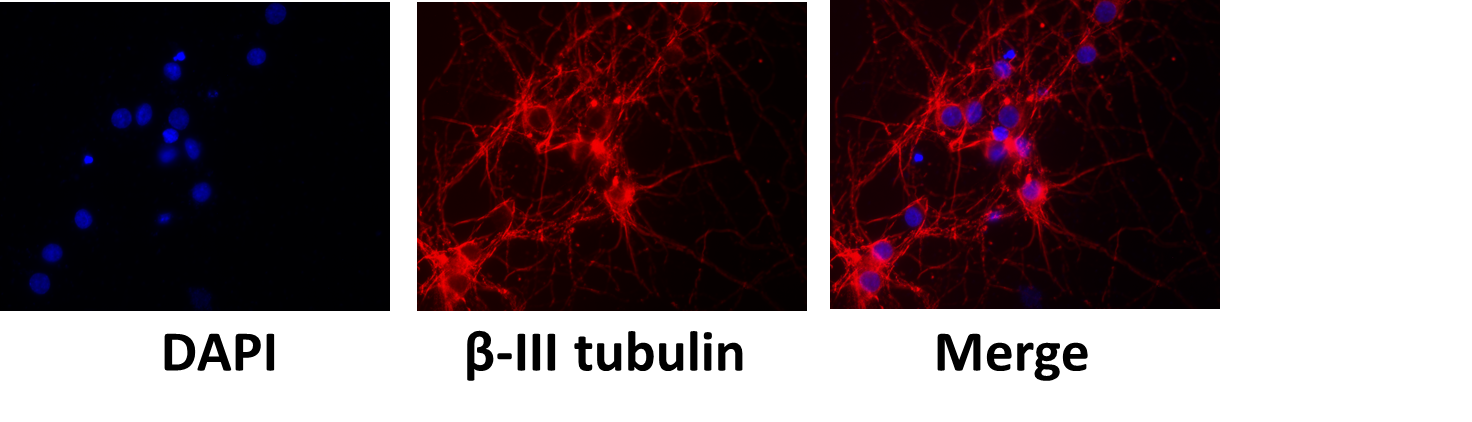


S1. Primary rat cortical culture was stained with the neuronal marker, β-III tubulin (red) to clarify the purity of neurons in the culture. More than 90% of DAPI (blue) stained nuclei were positive with β-III tubulin in the merge pictures, which indicated a highly purified culture.

S2. The dose-dependent response of 6-OHDA induced apoptosis and cytotoxicity and the protection of meclizine on primary rat cortical culture

S2A. Representative images of Fluoro-Jade C (FJ-C) staining on 6-OHDA induced cell death. No-toxin treatment caused few detectable FJ-C-stained neurons. Treatment with 10μM 6-OHDA for 24 hours resulted in more neurons with FJ-C stain (green) (arrow). Treatment with 20μM 6-OHDA for 24 hours remarkably increased the number of FJ-C-stained cells with nuclear morphological change (nuclear stained by DAPI, blue), which indicated the neurons underwent the process of death (arrowhead).


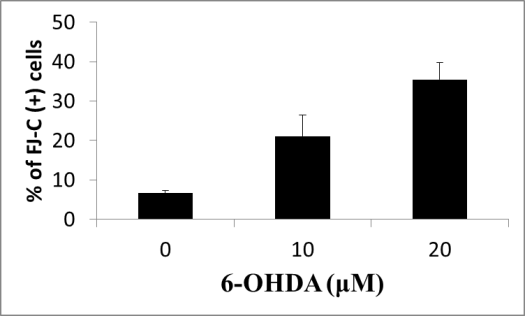


S2B. 6-OHDA induced a dose-dependent cytotoxicity on rat primary cortical culture cells. 10 μM of 6-OHDA treatment for 24 hours generated a remarkable but not overwhelming cell death (21.10±5.37% of FJ-C(+) cells, n=4). Data were presented as mean±S.E.M.


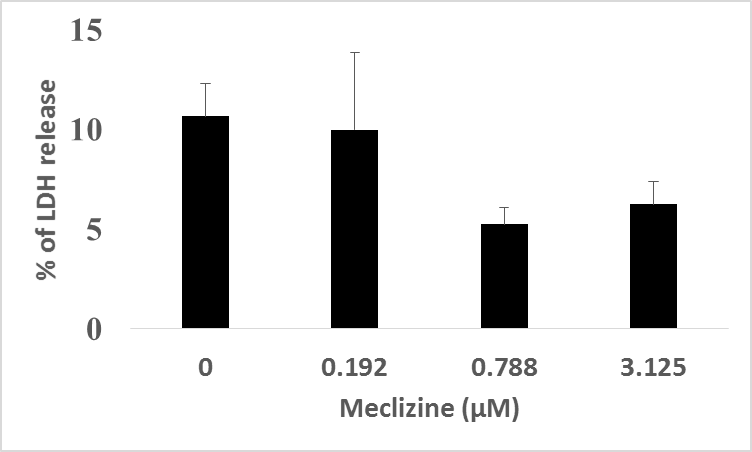
.

S2C. The protection of meclizine against 10μM 6-OHDA treatment for 24 hours was dose-dependent. There was no protection of 0.192μM of meclizine but 0.788 and 3.125μM meclizine remarkably reduced the cell death measured by LDH release assay.


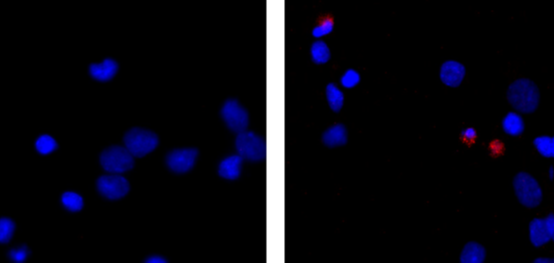
.

S2D. Representative images of cleaved caspase-3 immunostaining (red) to identify apoptotic cells in rat primary cortical cultures, against blue fluorescence DAPI nuclear staining of all cells. Spontaneous apoptotic cells were scant (left) whereas 20μM 6-OHDA treatment for 6 hours triggered more apoptotic cells as identified by red fluorescence (marked by arrowhead) (right).

S3. Meclizine did not protect 6-OHDA induced oxidative stress


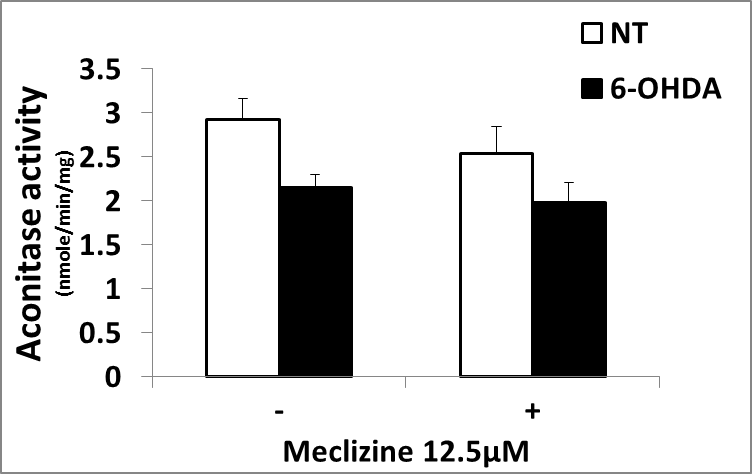


*

N.S.

N.S.

S3. 12.5μM meclizine, which demonstrated neuroprotection against 6-OHDA

induced cell death, did not significantly alter the aconitase enzymatic activity of SH-SY5Y cells in either no toxin or 30μM 6-OHDA treatment for 24 hours groups compared with control (no toxin: 2.9±0.2 from control versus 2.5±0.3 from 12.5μM meclizine, p>0.05, 30μM 6-OHDA: 2.2±0.1 from control versus 2.0±0.2nmole/min/mg from 12.5μM meclizine, p<0.05, n=10)(*, p<0.05, N.S., p>0.05).

S4. Meclizine affected neither oxidative phosphorylation nor cellular total ATP level


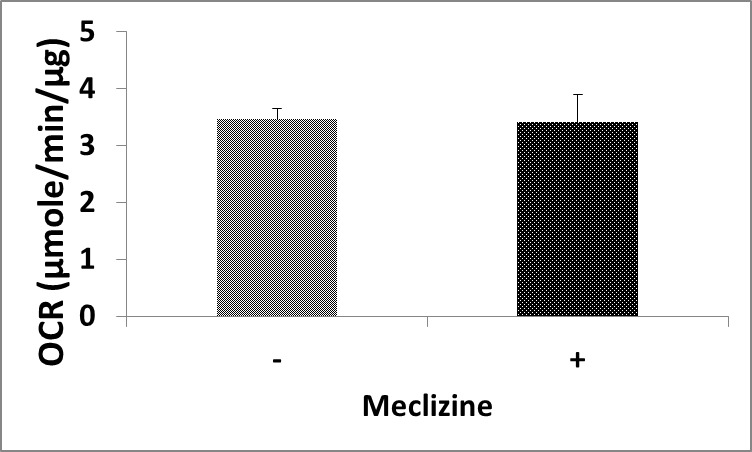


N.S.

S4A. 12.5μM meclizine treatment for 48 hours did not affect the oxygen consumption rate (OCR) measured by XF analyzer (control: 3.45±0.20, meclizine:3.41±0.48 μmole/min/μg, p>0.05, n=4).


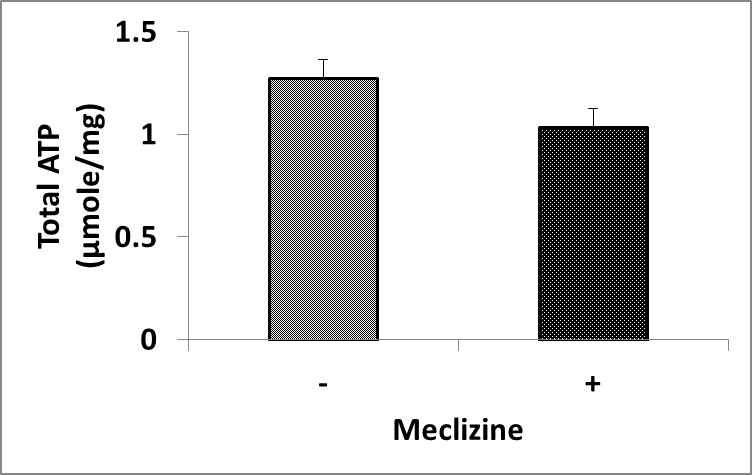


N.S.

S4B. 12.5μM meclizine treatment for 48 hours did not affect the total cellular ATP level (control: 1.27±0.09, meclizine:1.04±0.09 μmol/mg, n=10, p>0.05)

S5 Meclizine increased PFKFB3 protein amount in the rat primary cortical neurons


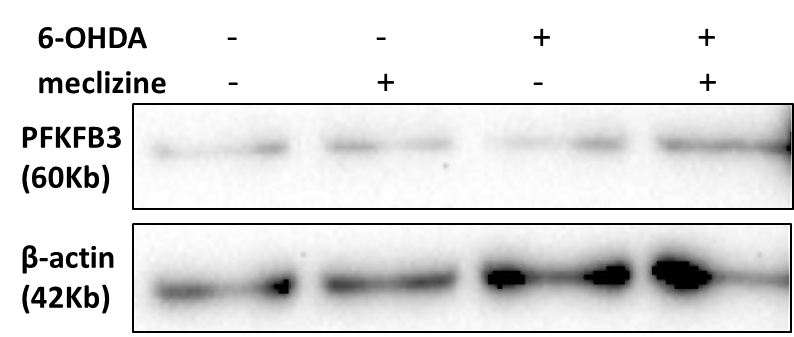


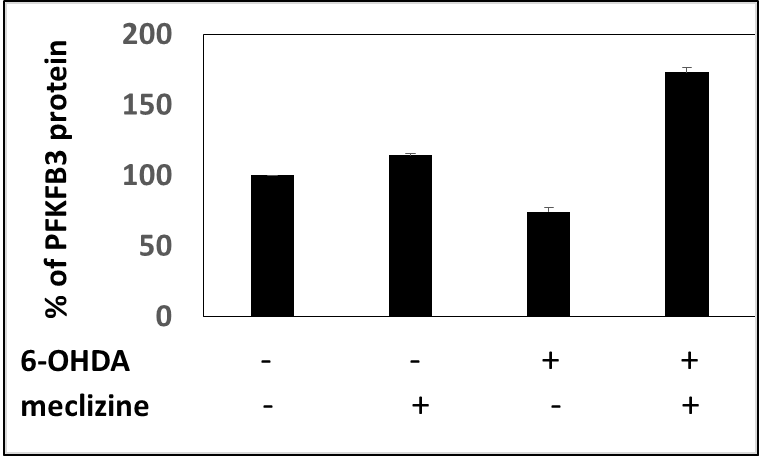


S5 Representative Western blot analysis and densitometry analysis of the PFKFB3 protein amount in the rat primary cortical neurons. Comparing with control, 1μM meclizine treatment for 24 hours increased the PFKFB3 protein amount up to 114±1%. 30μM 6-OHDA treatment for 2 hours reduced the PFKFB3 protein amount down to 74±3%. Pre-treatment with 1μM meclizine for 24 hours not only prevented the down-regulation of PFKFB3 by 6-OHDA but also up-regulated it up to 173±3%. (n=2, data were presented as mean±S.E.M., the percentage of protein amount was normalized by control).
